# Split nitrogen applications provide no benefit over a single well timed application in rainfed winter wheat

**DOI:** 10.3389/fpls.2025.1698494

**Published:** 2025-11-06

**Authors:** Samson Olaniyi Abiola, Joao Luis Bigatao Souza, Raedan Sharry, Jolee R. Derrick, Meryem Maatougui, Daryl Brian Arnall

**Affiliations:** 1 Department of Plant Soil Sciences, Oklahoma State University, Stillwater, OK, United States; 2 Department of Land Resources and Environmental Sciences, Montana State University, Bozeman, MT, United States; 3 Agricultural Innovation and Technology Transfer Center (AITTC), College of Agriculture & Environmental Sciences (CAES), UM6P - Mohammed VI Polytechnic University, Ben Guerir, Morocco

**Keywords:** application timing, winter wheat, growing degree days, fertilizer management, agronomic practices

## Abstract

Optimizing nitrogen (N) fertilization strategies in winter wheat requires understanding the combination of application timings and rates. This is crucial for achieving the dual objectives of maximizing grain yield while maintaining adequate grain protein concentration (GPC). Research has been conducted in central Great Plains to document the value of a single delayed application on N. However, most of this work was performed at sub-optimum N rates, and without a comparison with split application. This study evaluated N fertilization across multiple site-years to identify optimal timing of split applications and single applications under sub-optimum and excessive N rates. Field experiments were conducted at three locations in Oklahoma across three growing seasons (2018-2021), testing two N rates (100 and 200 kg N ha⁻¹) applied at five timing intervals based on growing degree days (0, 30, 60, 90, and 120 GDD). The split application received 50 kg pre- and 50 kg in season. The 100 kg rate was chosen to represent sub-optimum, while the 200 kg rate was in excess. The 100 kg N ha⁻¹ achieved grain yields of 5.0 Mg ha⁻¹, statistically similar to 200 kg N ha⁻¹ (5.1 Mg ha⁻¹) across all application timings, but GPC was increased at all timings except 120 GDD. These results confirm that 100 kg N ha⁻¹ was sub-optimum for protein but adequate for yield, while 200 kg N ha⁻¹ was excessive for yield but achieved premium protein. Application timing significantly influenced both yield and GPC, with 90 GDD applications producing optimal yields (5.4 Mg ha⁻¹) and adequate protein levels (12.5% GPC). In-season applications at 30–90 GDD achieved 12% higher GPC compared to pre-plant applications, while maintaining equivalent yields at 11.6%. Early applications (0–90 GDD) sustained maximum yield potential. Late applications (120 GDD) increased GPC by 1.2%. In-season applications aligned with crop demand periods which could potentially reduce operational costs and time compared to split applications by eliminating the need for multiple tractor operations. These results demonstrate the importance of strategic timing over increased N rates for achieving sustainable wheat production that balances high yields, adequate protein levels, and efficient N use while minimizing environmental impacts.

## Introduction

Winter wheat (*Triticum aestivum* L.) is an important cereal crop providing more than 25% of the grain protein concentration (GPC) to 40% of the global population and supporting agricultural economies across diverse production regions (Food and Agriculture Organization of the United Nations, 2015; FAOSTAT, 2018; Singh et al., 2023a). In the Great Plains of North America, winter wheat production faces the dual challenge of maximizing grain yield while maintaining adequate GPC. Especially under increasingly variable environmental conditions and economic pressures (Zhao et al., 2022). The optimization of nitrogen (N) fertilization strategies has emerged as a critical determinant of sustainable wheat production, requiring precise management that balances economic viability with environmental stewardship (Cassman et al., 2002; Snyder, 2017).

The relationship between N application timing, rates, and wheat performance represents one of the most complex agronomic challenges in cereal production systems. Current fertilization practices often exceeds crop physiological requirements, resulting in reduced N use efficiency (NUE), increased production costs, and environmental risks including nitrate leaching and greenhouse gas emissions (Noulas et al., 2023; Wang et al., 2011). Excessive N can also reduce grain yield through lodging, delayed maturity, and increased disease susceptibility, creating a counterproductive cycle that undermines both economic and environmental objectives (Li et al., 2022; Huang et al., 2021).

The physiological basis for optimizing N management lies in understanding the temporal dynamics of crop N demand relative to soil N availability. Winter wheat exhibits distinct phases of N uptake, with critical periods occurring during the tillering, stem elongation, and grain filling stages (Alcoz et al., 1993; Barneix, 2007; Szczepaniak et al., 2022). The synchronization of N supply with these physiological demand periods represents a fundamental principle for maximizing NUE. This approach enables achieving dual yield and GPC objectives. However, the practical implementation of this concept requires validation across diverse environmental conditions. This validation is crucial for developing robust management recommendations for various production systems.

The predictive accuracy of low-rate N responses for higher application rates represents a critical knowledge gap. Moreover, this gap has significant implications for fertilizer research methodology and practical recommendations. If response patterns observed at moderate N rates accurately predict agronomic behavior at higher rates, this would enable more efficient and cost-effective fertilizer research. Additionally, this approach would reduce the risk of environmental contamination from excessive N applications (Pan et al., 2020). However, most previous studies were conducted at sub-optimum N rates without comprehensive comparisons between split and single application methods (Singh et al., 2023b; Singh et al., 2024; Abiola et al., 2024; Ballagh et al., 2025). This limits the practical applicability of these findings to modern high yielding agricultural systems.

Recent advances in understanding wheat N physiology have revealed the importance of application timing in determining both yield and GPC outcomes. Studies have demonstrated that delayed N applications could enhance GPC without compromising yield (Miller et al., 2017; Souza et al., 2022), suggesting that timing strategies could overcome the traditional yield-GPC trade-off (Wu et al., 2025). However, application timing effects involve intricate interactions between physiological development stages, environmental conditions, and N availability patterns. Traditional approaches often prioritize either yield maximization or GPC enhancement, leading to suboptimal outcomes when both objectives are considered simultaneously (Triboi et al., 2006; Sieling and Kage, 2021). Therefore, identifying application timings that optimize both yield and GPC outcomes is crucial for providing producers with improved management strategies.

To address this complexity, the use of growing degree days (GDD) as a physiological timing indicator may offer advantages over calendar-based applications by accounting for temperature-driven developmental processes (Oklahoma Mesonet Station, 2012). However, optimal GDD timing for different N management objectives requires testing across multiple environments and growing seasons. This comprehensive evaluation is essential because traditional approaches often prioritize either yield maximization or GPC enhancement, leading to suboptimal outcomes when both objectives are considered simultaneously (Triboi et al., 2006; Sieling and Kage, 2021).

On the other hand, the concept of split N applications has gained attention as a potential strategy for improving NUE by better matching N supply with crop demand patterns (Mohammed et al., 2013; Preza-Fontes et al., 2021). The advantages include reduced N losses through leaching and volatilization, improved N uptake efficiency during critical growth stages, and enhanced flexibility in responding to seasonal weather variations (Schulz et al., 2015; Ma et al., 2021). However, empirical evidence supporting these benefits remains inconsistent, particularly when comparing split applications to strategically timed single applications under varying environmental conditions (Tedone et al., 2018; Hu et al., 2021).

This study aims to provide a comprehensive understanding of N fertilization strategies in winter wheat production to inform management practices that optimize both grain yield and GPC. Particularly, focus on applying the 4R concept, specifically emphasizing the right rate and right timing of N application. The research addresses critical knowledge gaps in understanding the interactions between N rates, application timing, and split application under diverse central Great Plains environmental conditions. To address these gaps, our objectives were to (1) determine whether agronomic response patterns observed at sub-optimum N rates (100 kg N ha⁻¹) are consistent with those at high rates (200 kg N ha⁻¹), (2) assess how application timing influences grain yield and GPC relationships in winter wheat, and (3) investigate whether split N applications improve grain yield and GPC compared to single in-season applications.

We tested three hypotheses: (H1) relative agronomic response patterns to application timing will be consistent between sub-optimum (100 kg N ha⁻¹) and excessive (200 kg N ha⁻¹) N rates, (H2) application timing significantly influence both grain yield and GPC, and (H3) split N applications will improve grain yield and GPC compared to single in-season applications. These hypotheses address fundamental questions about N rate optimization, application method efficiency, and timing effects on dual yield-protein objectives in central Great Plains winter wheat production systems.

## Materials and methods

### General experiment information

A non-irrigated research study was conducted across three sites in Oklahoma from 2018 to 2021. Specifically, experiments were conducted at three sites during the 2018–2019 and 2019–2020 seasons, with two sites utilized for the 2020–2021 season. Research sites included the Ballagh family research farm (36°52’N, 97°03’W, Newkirk), Oklahoma Research Stations at Lake Carl Blackwell (LCB) (36°09’N, 97°14’W), and Perkins (35°59’N, 97°02’W). All experimental sites were managed under no-till practices. The soil type at Ballagh was Agra-Foraker soil (Fine, mixed, superactive, thermic Udertic Paleustolls). At LCB, the soil was Pulaski soil (Coarse-loamy, mixed, superactive, nonacid, thermic Udic Ustifluvents), and in Perkins was Teller series (fine loamy, mixed, active, thermic Udic Argiustolls) and Konawa series (fine loamy, mixed, active, thermic Ultic Haplustalfs) (USDA/NRCS soil taxonomy). Weather information was acquired daily from planting to harvest (i.e., from October to June) from automated weather stations operated by the Mesonet Oklahoma weather network (McPherson et al., 2007), located proximately to the research sites (**Table 1**).

**Table 1:** Weather information for each growing season, site, and season showing cumulative precipitation (Cum PPT), 10-year average cumulative precipitation (10-yr Cum PPT), maximum temperature (T max), minimum temperature (T min), average temperature (T avg), 10-year average maximum temperature (10-yr T max), 10-year average minimum temperature (10-yr T min), 10-year average temperature (10-yr T avg), and cumulative growing degree days (Cum GDD) across three sites during 2018-2019, 2019-2020, and 2020–2021 growing seasons.

Growing season	Site	Season	Cum PPT	10-yr Cum PPT	T max	T min	T avg	10-yr T max	10-yr T Min	10-yr T Avg	Cum GDD
			——mm——–		—————————————°c——————————————						
2018-2019	Ballagh	Fall	2.79	6.35	9.91	-2.66	3.45	15.49	2.68	8.78	152.84
		Winter	5.59	3.30	8.90	-2.21	3.26	11.66	-1.53	4.81	422.69
		Spring	23.11	14.48	24.96	13.60	19.12	25.67	13.55	19.55	1754.4
	LCB	Fall	3.30	5.59	11.23	-2.75	3.94	8.66	-5.78	1.34	250.24
		Winter	6.35	3.81	10.53	-1.45	4.48	10.15	-4.41	2.85	483.68
		Spring	24.64	15.75	25.86	13.32	19.65	12.32	-0.62	6.02	1782.92
	Perkins	Fall	9.40	6.86	14.55	2.84	8.39	16.67	4.21	10.18	796.18
		Winter	6.35	4.57	10.18	-0.26	4.82	11.87	-0.97	5.31	511.48
		Spring	25.91	14.99	25.87	14.17	19.80	22.65	10.59	16.53	1822.06
2019-2020	Ballagh	Fall	2.032	6.35	11.38	-0.84	4.70	15.74	2.79	8.95	208.80
		Winter	8.13	3.56	12.79	0.26	6.23	11.37	-1.77	4.56	625.76
		Spring	6.86	13.72	25.50	12.79	18.99	25.72	13.51	19.55	1742.58
	LCB	Fall	2.29	6.10	13.30	-1.00	5.61	8.91	-5.73	1.48	346.03
		Winter	8.64	4.32	14.20	0.60	7.25	9.85	-4.71	2.56	687.45
		Spring	5.59	15.24	26.40	12.38	19.62	12.32	-0.67	5.99	1764.53
	Perkins	Fall	7.37	7.11	15.60	2.59	8.68	16.75	4.18	10.19	825.53
		Winter	10.41	5.33	14.09	2.08	7.82	12.15	-0.59	5.62	746.20
		Spring	6.86	13.97	26.31	13.62	19.93	23.11	10.97	16.96	1816.67
2020-2021	LCB	Fall	3.81	6.10	14.26	-0.51	6.66	9.16	-5.58	1.69	388.98
		Winter	6.60	4.32	11.29	-2.10	4.73	9.99	-4.71	2.66	562.46
		Spring	12.70	14.73	25.12	12.75	18.93	12.13	-0.86	5.79	1723.20
	Perkins	Fall	8.89	6.86	17.24	3.94	10.13	17.11	4.36	10.45	962.91
		Winter	5.33	5.33	11.21	-0.41	5.35	12.05	-0.57	5.59	617.33
		Spring	19.05	14.48	25.20	13.60	19.25	23.34	11.27	17.21	1765.70

Fall: October to December; Winter: January to March; Spring: April to June.

Winter wheat varieties adapted to Oklahoma growing conditions were used consistently within each site-year. Soil fertility was evaluated at the time of sowing for each site-year (**Table 2**). Composite soil samples were collected at a depth of 0–15 cm prior to sowing for each site-year. Soil samples were analyzed at the Oklahoma State University Soil, Water, and Forage Analytical Laboratory. The soil pH was determined using a 1:1 soil–to–water ratio. The buffer index was measured using the Sikora method. Nitrate-N was extracted with 1M KCl. Phosphorus, potassium, calcium, and magnesium were extracted using Mehlich-3 solution. Sulfate was determined using a calcium phosphate extraction method. Organic matter (OM) was determined by loss on ignition. Phosphorus and potassium fertilization were managed according to Oklahoma State University Extension recommendations based on soil test results (Warren et al., 2017). Diseases, insects, and weeds were chemically controlled as needed, in accordance with recommendations from the Oklahoma State University Extension.

**Table 2:** Soil fertility information at sowing (0–15 depth) showing soil pH, organic matter (OM), buffer index (BI), nitrate nitrogen (NO_3_), Mehlich-3 extractable phosphorus (M3P), potassium (K), sulfate (SO_4_), calcium (Ca), and magnesium (Mg) across three sites (Ballagh, LCB, and Perkins) during 2018-2019, 2019-2020, and 2020–2021 growing seasons.

Growing seasons	Site	pH	OM	Bl	NO_3-_	M3P	K	SO_4_	Ca	Mg
			%	———————– mg kg^-1^—————————						
2018-2019	Ballagh	6.70	3.15		9.50	31.50	94.50	4.00	2421.50	229.50
	Perkins	4.60	0.89	6.40	3.50	24.50	130.00	4.50	356.00	100.00
	LCB	5.90	1.72	7.10	4.00	25.00	112.50	3.00	1314.00	221.00
2019-2020	Ballagh	7.30	2.63		2.50	20.50	104.50	9.70	2595.00	290.00
	Perkins	4.80	0.92	6.50	6.00	26.00	140.50	7.17	386.50	98.50
	LCB	5.20	1.47	7.10	9.00	26.50	89.00	3.35	709.50	154.50
2020-2021	Perkins	6.80	0.80		1.00	51.00	147.00	2.24	644.00	137.50
	LCB	5.50	0.81	7.20	12.50	19.50	73.00	3.80	690.00	120.00

### Experimental design and treatment structure

The experiment was arranged in a randomized complete block design (RCBD) with 15 treatments and four replications. Individual plots measured 3.1 m × 6.1 m, with alleys of 3.1 m between plots, resulting in a total trial area of 34 m × 49 m. Top-dress N applications were made at different growing degree days (GDD > 0) timings: 30, 60, 90, or 120 GDD. The treatment consisted of various combinations of N application rates and timings (**Table 3**). Three applications included Pre-plant N, in-season N, and split application at different N rates of 0, 50, 100, or 200 kg N ha⁻¹ (**Table 4**). For each experimental plot, 50 kg N ha⁻¹ of ammonium nitrate was applied at the specified GDD timings. This design enabled direct comparison of split versus single applications while identifying optimal timing across sub-optimum and excessive N rates.Growing degree day calculation and rationale.

**Table 3:** Planting and nitrogen application dates based on growing degree days (GDD ≥ 0) at 0, 30, 60, 90, and 120 GDD across three sites (Ballagh, LCB, and Perkins) during 2018-2019, 2019-2020, and 2020–2021 growing seasons.

Growing seasons	Site	0	30	60	90	120
		————————————–GDD————————————				
2018-2019	Ballagh	11-24-2018	12-18-2018	03-07-2019	04-12-2019	05-14-2019
	Perkins	10-03-2018	11-29-2018	12-20-2018	02-19-2019	04-03-2019
	LCB	11-07-2018	1-22-2019	03-15-2019	04-15-2019	05-07-2019
2019-2020	Ballagh	10-18-2019	11-27-2019	02-05-2020	03-23-2020	04-21-2020
	Perkins	11-19-2019	11-26-2019	12-30-2019	02-17-2020	03-23-2020
	LCB	10-15-2019	11-27-2019	01-24-2020	03-10-2020	04-09-2020
2020-2021	Perkins	10-18-2020	11-15-2020	01-04-2021	03-04-2021	04-03-2021
	LCB	10-14-2020	11-15-2020	01-04-2021	03-04-2021	04-03-2021

**Table 4:** Nitrogen treatment structure showing pre-plant, in-season, split-applied, N application rates, total N applied and top-dress timing (kg N ha^-1^) based on growing degree days (GDD > 0).

Treatments numbers	Application	Pre-N	In season	Total N	Top-dress timing (GDD≥0)
		————kg N ha^-1^———–			
1	Control	0	0	0	0
2	Pre-plant	200	0	200	0
3	In-season	0	200	200	30
4	In-season	0	200	200	60
5	In-season	0	200	200	90
6	In-season	0	200	200	120
7	Pre-plant	100	0	100	0
8	In-season	0	100	100	30
9	In-season	0	100	100	60
10	In-season	0	100	100	90
11	In-season	0	100	100	120
12	Split-applied	50	50	100	30
13	Split-applied	50	50	100	60
14	Split-applied	50	50	100	90
15	Split-applied	50	50	100	120

Application timing was based on growing degree days (GDD > 0) rather than calendar dates to account for temperature-driven developmental processes and improve consistency across environments. The use of GDD as a physiological timing indicator offers advantages over calendar-based applications by synchronizing N supply with crop developmental stages regardless of seasonal weather variations (Oklahoma Mesonet Station, 2012). GDD > 0 was calculated using the Oklahoma Mesonet Station (2012) cutoff method (Equation 1):

(1) $$1\text{ GDD}>0=\frac{\left(\text{Day Max Temperature}+\text{Day Min Temperature}\right)}{2}-4.4\;{}^{\circ}\text{C}$$

### Grain yield and protein measurements

Grain yield was harvested at maturity from the center 1.5 m of each plot using a Kincaid8-XP plot combine (Kincaid Equipment Manufacturing, Haven, KS, United States). Grain yield and moisture content were recorded by the onboard Harvest Master Yield monitoring computer (Juniper Systems, Logan, UT, United States). Grain moisture content was adjusted to 12.5% to standardize yield measurements. Grain protein concentration was determined using near-infrared spectroscopy with a Diode Array NIR Analysis System model DA 7200 (Perten, Kungens Kurva, Sweden).

### Statistical analysis

Prior to statistical analysis, we evaluated the homogeneity of variances using Levene’s tests from the ‘car’ package to evaluate whether data could be combined across locations and years for both grain yield and GPC (Fox et al., 2012). Linear mixed models were applied to evaluate the treatment effects on measured parameters. For each parameter at each location, we fitted models considering N treatment as a fixed effect and blocks nested within each site-year as a random effect.

Data was analyzed using three distinct approaches to address specific research objectives. The first analysis evaluated N rate effects (0, 100, and 200 kg N ha⁻¹) within each GDD timing (0, 30, 60, 90, and 120 GDD) for both grain yield and GPC. The second analysis compared GDD timing effects within each N rate to determine optimal application timing. The third analysis compared applications (pre-plant, in-season, and split application) at each GDD timing (30, 60, 90, and 120 GDD) while maintaining a constant total N rate of 100 kg N ha⁻¹.

For each analysis, mean comparisons were performed using Tukey’s honestly significant difference (HSD) test at α = 0.05. Statistical analyses were conducted using R software version 4.1.2 with packages “lme4” (Bates et al., 2015), “lmerTest” (Kuznetsova et al., 2017), “multcomp” (Hothorn et al., 2008), “dplyr” (Wickham et al., 2020), and “ggplot2” (Wickham, 2016).

## Results

Levene’s test revealed significant heterogeneity of variances among site-years for both grain yield and GPC (P < 0.05) (**Supplementary Figure 1**). However, treatment response patterns remained consistent for each site-year analysis. Similarly, combined analysis across site-years produced the same trends as individual site-year analyses. Therefore, results are presented as averages across all site-years to provide a comprehensive overview of treatment effects, with detailed site-year data available in **Supplementary Tables 1**–**3**.

### Nitrogen rate (100 vs 200 kg N ha⁻¹) effect on grain yield and protein concentration

Grain yield responses demonstrated consistent patterns between 100 and 200 kg N ha⁻¹ rates across all application timings. The 100 kg N ha⁻¹ rate achieved 4.5 Mg ha⁻¹ while the 200 kg N ha⁻¹ rate produced 4.6 Mg ha⁻¹ (**Table 5**). Both rates increased yields significantly from the unfertilized control (2.5 Mg ha⁻¹) to near-maximum potential. Individual site-year yields ranged from 1.7 to 7.0 Mg ha⁻¹ for the 100 kg N ha⁻¹ treatment and from 2.6 to 6.8 Mg ha⁻¹ for the 200 kg N ha⁻¹ treatment. This yield equivalency was maintained across 80% of timing applications (4 of 5 GDD timings per site-year) (**Supplementary Table 1**). Individual site-year yields ranged from 0.8 to 4.5 Mg ha⁻¹ for the control, 1.7 to 7.0 Mg ha⁻¹ for 100 kg N ha⁻¹, and 2.6 to 6.8 Mg ha⁻¹ for 200 kg N ha⁻¹ treatments.

However, GPC responses revealed parameter-specific predictive limitations. While timing response patterns remained identical between rates, absolute GPC values diverged significantly. GPC increased from 12.4% to 14.2% when comparing 100 to 200 kg N ha⁻¹ at planting (0 GDD) (**Table 5**). Both rates achieved maximum GPC at 120 GDD (14.6% vs. 15.8% for 100 vs. 200 kg N ha⁻¹, respectively), demonstrating parallel response curves despite the absolute value differences.

**Table 5:** Grain yield and grain protein concentration response to nitrogen application rates across different application timings in winter wheat across all site-years.

N rates	0		30		60		90		120	
	Yield	GPC	Yield	GPC	Yield	GPC	Yield	GPC	Yield	GPC
0	2.5b	11.0c	2.5b	11.0c	2.5b	11.0c	2.5b	11.0c	2.5b	11.0b
100	4.5a	12.4b	4.2a	13.0b	4.5a	13.0b	4.3a	13.7b	3.4a	14.6a
200	4.6a	14.2a	4.6a	14.8a	4.6a	14.8a	4.3a	15.6a	3.4a	15.8a
*p-value*	*<0.01*	*<0.01*	*<0.01*	*<0.01*	*<0.01*	*<0.01*	*<0.01*	*<0.01*	*<0.01*	*<0.01*

Different letters represent the significant difference (Tukey, HSD) at the 0.05 probability level.

### Grain yield and protein concentration as influenced by nitrogen application timing

**Figure 1:**
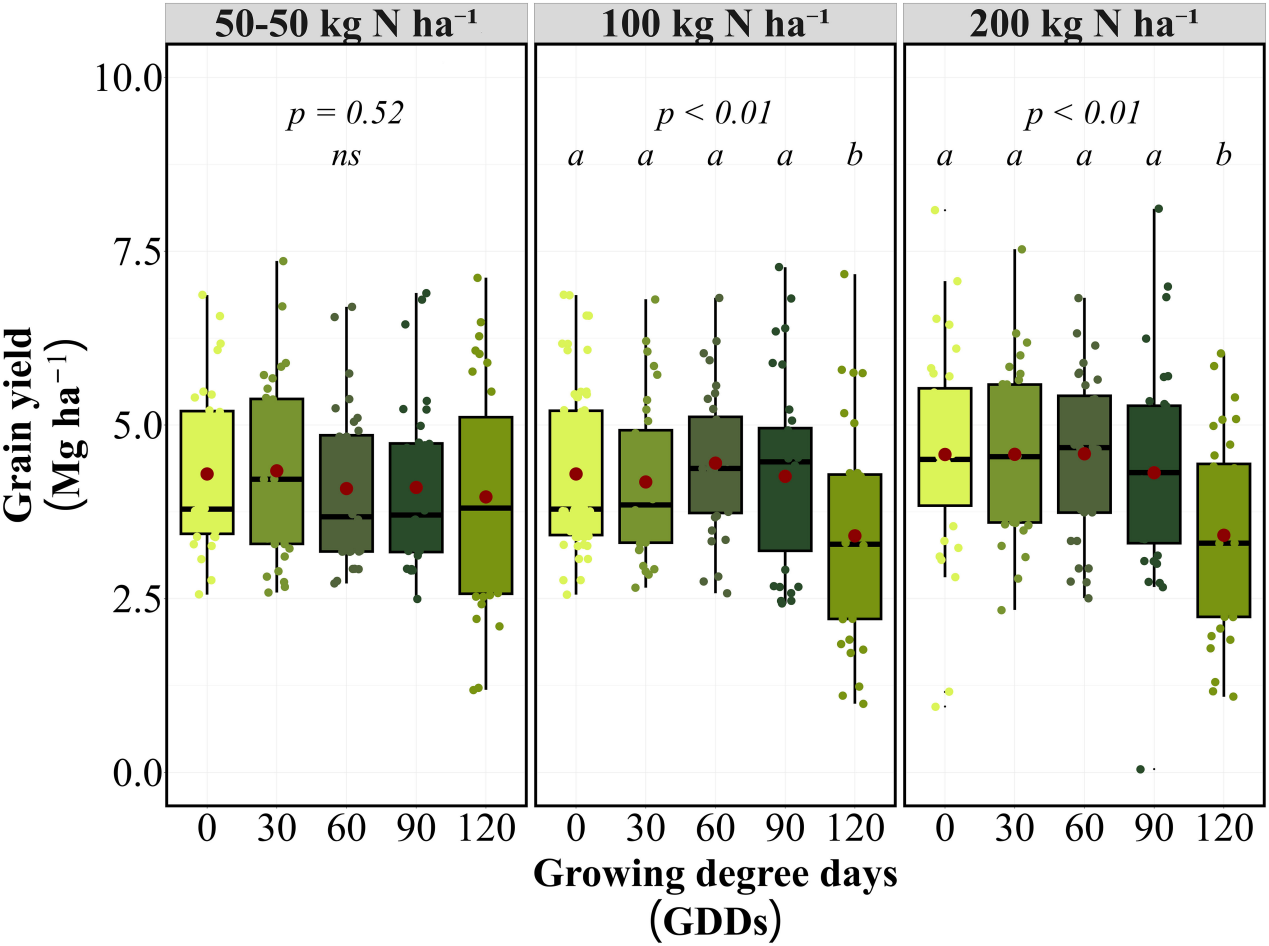
Grain yield (Mg ha^-1^) response to N application timing at 100 and 200 kg N ha⁻¹ rates on average of eight site-years. Box plots show median, quartiles, and range of grain yield values at different growing degree day (GDD) application timings. Red points indicate treatment means. Letters indicate significant differences among timing treatments within each N rate (p < 0.01).

For grain yield optimization, early applications (0–90 GDD) maintained maximum yield potential across both N rates (**Figure 1**). Split applications demonstrated timing-insensitive performance, with the 50–50 kg N ha⁻¹ split application showing no significant timing effect (*P = 0.52*) and consistent yields (4.2-4.8 Mg ha⁻¹) across all timings. At 100 kg N ha⁻¹, application at 0 GDD achieved the highest yield (4.5 Mg ha⁻¹), which was statistically equivalent to early in-season applications at 30, 60, and 90 GDD (4.2-4.5 Mg ha⁻¹). Late application at 120 GDD resulted in significantly lower yields (3.4 Mg ha⁻¹) compared to all earlier timings. Similar timing effects were observed at 200 kg N ha⁻¹, with applications at 0, 30, and 60 GDD producing equivalent yields (4.6 Mg ha⁻¹). The 90 GDD timing showed intermediate performance (4.3 Mg ha⁻¹), while 120 GDD again produced the lowest yields (3.4 Mg ha⁻¹).

The timing response pattern remained consistent between 100 and 200 kg N ha⁻¹ rates, with both rates showing similar relative performance across GDD timings. Environmental variability was most pronounced at the 120 GDD timing, where yield reductions ranged from 15% to 35% compared to other timings across site-years. The timing response pattern was consistent across individual site-years, with early applications (0–60 GDD) outperforming late applications (120 GDD) for yield in 7 of 8 site-years, regardless of N rate.

**Figure 2:**
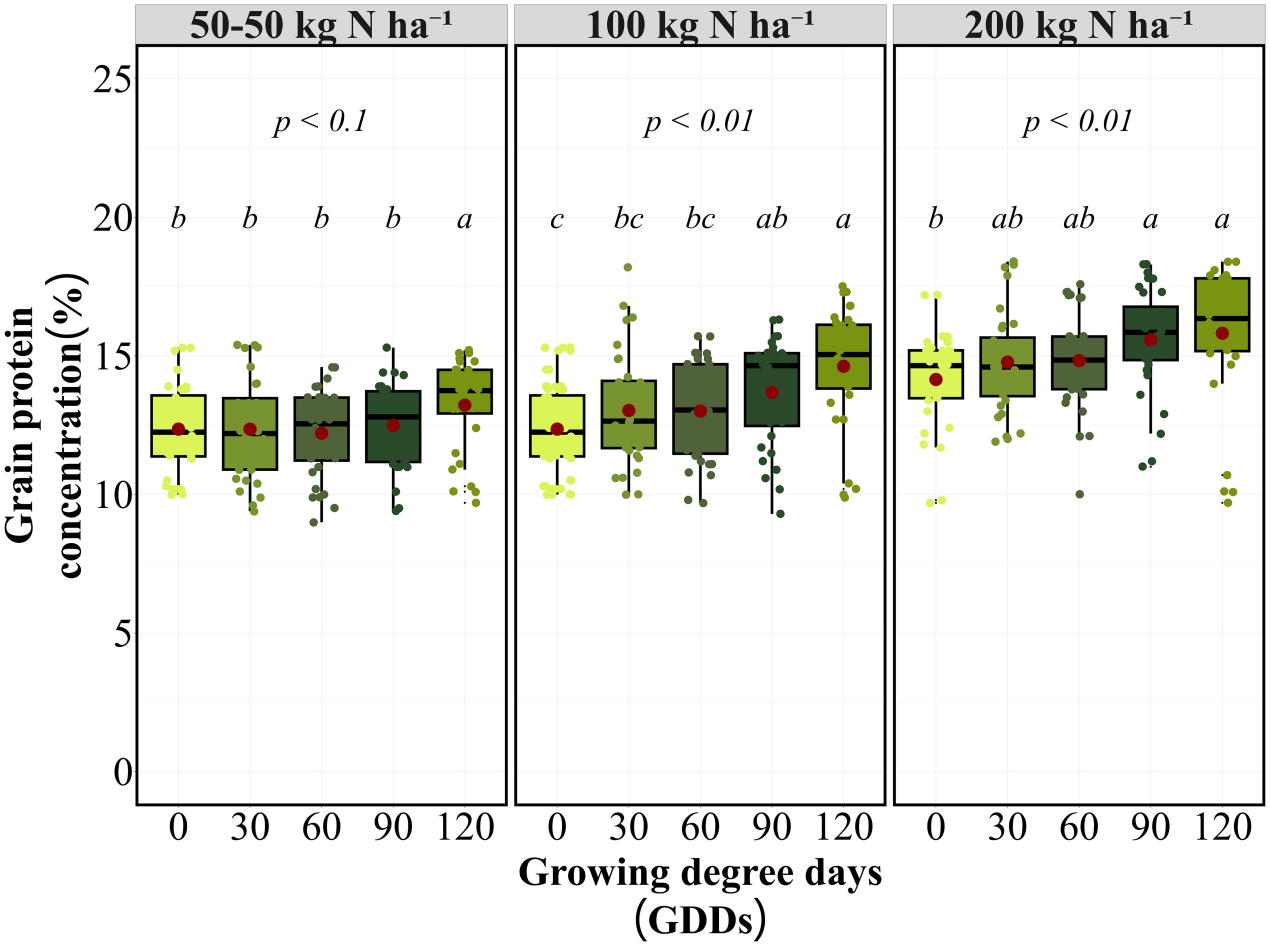
Grain protein concentration (%) response to N application timing at 100 and 200 kg N ha⁻¹ rates on average of eight site-years. Box plots show median, quartiles, and range of GPC values at different growing degree day (GDD) application timings. Red points indicate treatment means. Letters indicate significant differences among timing treatments within each N rate (p < 0.01).

For the GPC, timing effects varied by application strategy (**Figure 2**). Single applications at 100 and 200 kg N ha⁻¹ demonstrated highly significant timing effects *(P < 0.01)*, with GPC exhibiting an opposite response pattern to yield. Later applications produced significantly higher GPC levels, with GPC increasing from 12.4% at 0 GDD to 14.6% at 120 GDD for a 100 kg N ha⁻¹ application. At 200 kg N ha⁻¹, the timing effect followed the same pattern, with 120 GDD producing the highest GPC (15.8%), followed by 90 GDD (15.6%). The 90 GDD timing emerged as optimal for balancing yield and protein objectives, maintaining competitive yields while achieving substantial increases in GPC compared to earlier applications. At this timing, 100 kg N ha⁻¹ achieved 13.7% GPC with 4.3 Mg ha⁻¹ yield, while 200 kg N ha⁻¹ achieved 15.6% GPC with equivalent yield (4.3 Mg ha⁻¹). Conversely, GPC consistently increased with delayed applications in 6 of 8 site-years, demonstrating a clear yield-protein trade-off except at 90 GDD, where both parameters remained favorable.

The 90 GDD timing demonstrated an optimal balance between yield and protein objectives, maintaining competitive yields while achieving substantial increases in GPC compared to earlier applications. The timing response pattern was consistent across individual site-years, with early applications (0–60 GDD) outperforming late applications (120 GDD) for yield in 7 of 8 site-years, regardless of N rate (**Supplementary Table 2**). GPC consistently increased with delayed applications in 6 of 8 site-years, demonstrating a clear yield-protein trade-off except at 90 GDD, where both parameters remained favorable.

### Split versus single nitrogen application efficiency

**Figure 3:**
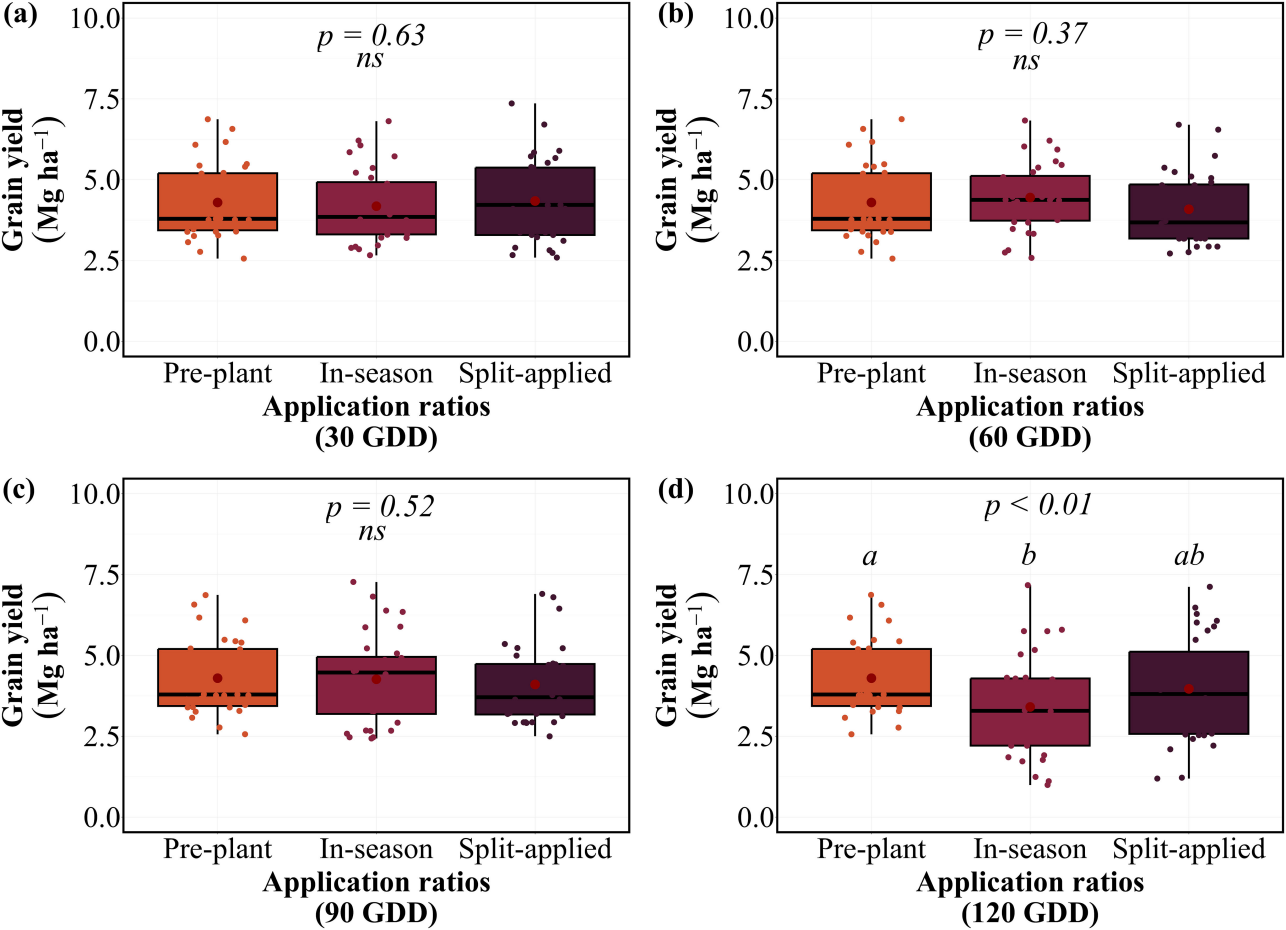
Grain yield response to N application at different GDD timings on average of eight site-years. Box plots show median, quartiles, and range of grain yield values for pre-plant, in-season, and split-applied treatments at **(a)** 30 GDD, **(b)** 60 GDD, **(c)** 90 GDD, and **(d)** 120 GDD. Red points indicate treatment means. Letters indicate significant differences among application within each timing (p < 0.01); ns indicates non-significant differences.

The application of N in a single dose or split applications effect on grain yield at equivalent total N rates of 100 kg N ha⁻¹ varied by GDD timing (**Figure 3**). Split applications (50 kg pre-plant + 50 kg in-season) demonstrated similar performance compared to single applications, with no clear efficiency advantages over single in-season applications. At 30 GDD, no significant differences were found among pre-plant (4.5 Mg ha⁻¹), split application (4.3 Mg ha⁻¹), and in-season application (4.2 Mg ha⁻¹). Similarly, at 60 and 90 GDDs, pre-plant (4.5 Mg ha⁻¹), in-season (4.5 Mg ha⁻¹), and split applications (4.1 Mg ha⁻¹) produced statistically equivalent yields. Significant differences emerged only at 120 GDD (*P < 0.01*), where pre-plant application (4.5 Mg ha⁻¹) significantly outperformed in-season application (3.4 Mg ha⁻¹), while split application showed intermediate performance (4.0 Mg ha⁻¹). The application response was consistent across site-years, with pre-plant applications maintaining stable performance in 7 of 8 site-years, regardless of comparison timing (**Supplementary Table 3**). Split applications demonstrated similar consistency in 6 of 8 site-years, while in-season variability increased with delayed comparison timings across environments.

**Figure 4:**
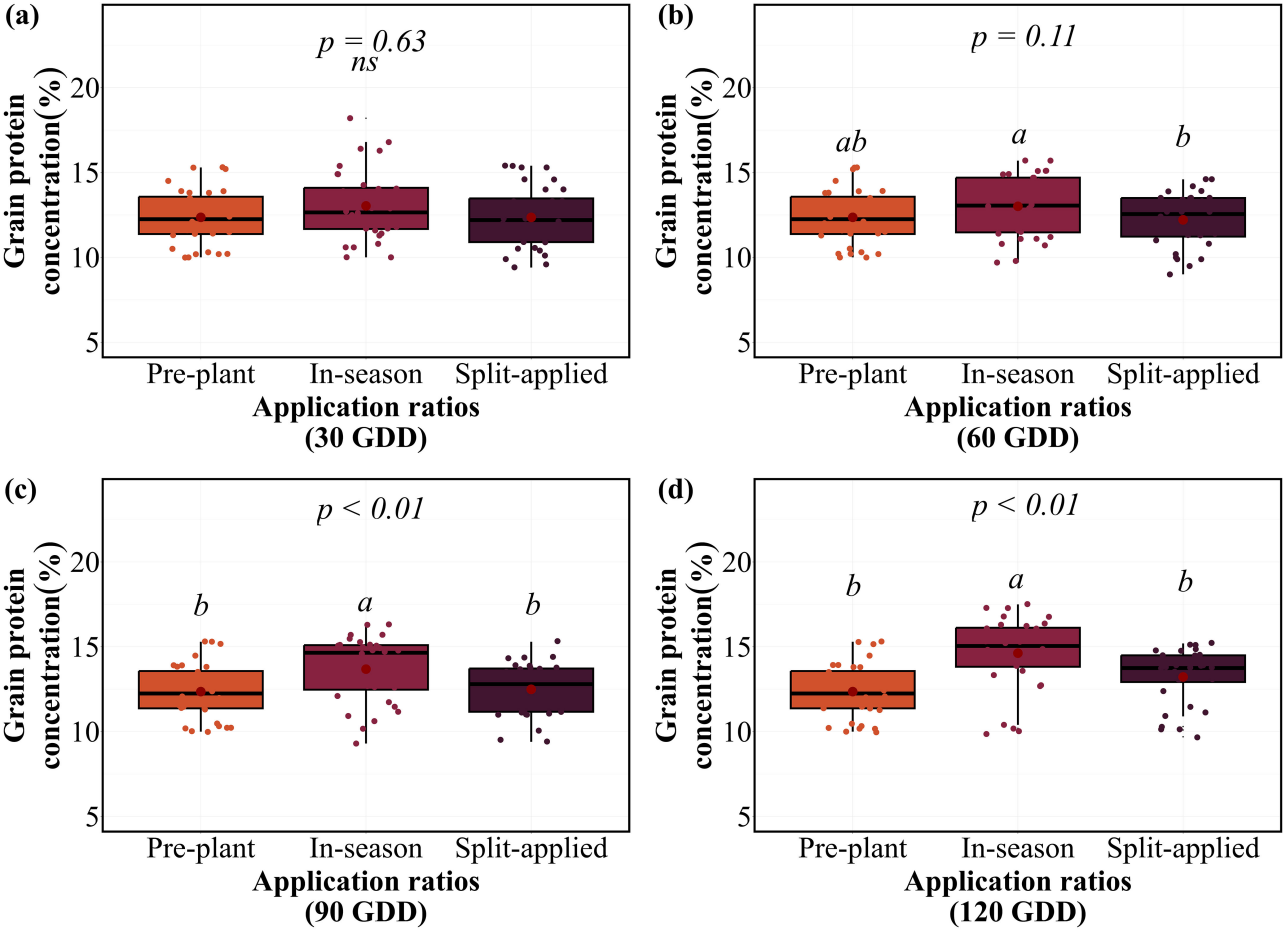
Grain protein concentration response to N application at different GDD timings on average of eight site-years. Box plots show median, quartiles, and range of grain yield values for pre-plant, in-season, and split-applied treatments at **(a)** 30 GDD, **(b)** 60 GDD, **(c)** 90 GDD, and **(d)** 120 GDD. Red points indicate treatment means. Letters indicate significant differences among applications within each timing (p < 0.01); *ns* indicates non-significant differences.

Grain protein concentration responses to the application varied with timing comparison (**Figure 4**). At 30 GDD, no significant differences were observed among applications, with GPC ranging from 12.2-13.0% across all applications. However, significant ratio effects emerged at 90 GDD (*P < 0.01*) and a tendency at 60 GDD (*P = 0.11*), where in-season application achieved significantly higher GPC (13.7%) compared to both split application (12.5%). This pattern intensified at 120 GDD, where in-season application produced the highest GPC (14.6%), followed by split application (13.2%), while pre-plant application maintained the lowest GPC (12.4%). In-season applications demonstrated superior efficiency at 60, 90, and 120 GDD timings. They achieved equivalent yields while producing higher GPCs compared to pre-plant applications. However, this advantage was lost at 120 GDD, where yield penalties outweighed GPC benefits. The application response was consistent across site-years, with in-season applications providing optimal yield-protein balance at early to mid-season timings in 6 of 8 site-years (**Supplementary Table 3**).

## Discussion

The agronomic performance of winter wheat under strategically designed N management strategies reveals critical insights into fertilizer response patterns. Our multi-environmental investigation across eight site-years was specifically designed to address a fundamental knowledge gap. Most previous research on delayed N applications was conducted at sub-optimum rates without a comprehensive comparison to split application strategies. By deliberately testing both sub-optimum (100 kg N ha⁻¹) and excessive (200 kg N ha⁻¹) N rates across five physiologically relevant timing windows. We provide among the first comprehensive framework for understanding N response patterns under Great Plains conditions.

We fail to reject our first hypothesis, as relative response patterns at 100 kg N ha^-1^ were consistent with those at 200 kg N ha^-1^ across timing, indicating the use of moderate rates for timing optimization studies. Both rates exhibited identical relative performance across all application timings. This finding validates the use of moderate rates for initial timing optimization studies. We also fail to reject our second hypothesis, as application timing significantly influenced yield-protein relationships, with in-season applications at 90 GDD emerging as the optimal physiological window for balancing dual agronomic objectives. However, we reject our third hypothesis regarding the advantages of split applications. Single in-season applications consistently outperformed split applications across multiple timing comparisons. This demonstrates that dividing N inputs provides no efficiency benefits when total rates are equivalent. These findings demonstrate that strategic timing precision may be the primary driver of NUE in central Great Plains dryland winter wheat production systems.

### Agronomic response to N rate

The consistency of response patterns between 100 and 200 kg N ha^-1^ rates addresses a critical knowledge gap in central Great Plains wheat production. We designated these rates as sub-optimum and excessive based on their relationship to dual production objectives, with the 100 kg N ha⁻¹ rate achieving maximum yield potential (4.5 Mg ha⁻¹) statistically similar to 200 kg N ha⁻¹ but produced GPC (12.4%) below typical market premiums (≥13.5% for hard red winter wheat in Oklahoma; USDA-Agricultural Marketing Service (USDA-AIMS, 2024). Conversely, the 200 kg N ha⁻¹ rate exceeded agronomic requirements for yield while achieving premium protein levels (14.2%), representing an excessive application from an economic and environmental perspective. The 1.8% protein enhancement at the higher rate indicates that crop N demand for quality objectives was not fully satisfied at 100 kg N ha⁻¹, despite yield saturation.

Most previous timing research has been conducted in the central Great Plains at moderate application levels without validation across the full fertilization range (Bushong et al., 2014; Souza et al., 2022; Singh et al., 2024; Abiola et al., 2025). Our multi-environment investigation demonstrates that agronomic response patterns at sub-optimum N rates were consistent with those at higher rates with confidence to commercial scenarios. This approach could reduce experimental costs and environmental N inputs while maintaining research validity. Cross validation analysis across eight site-years confirmed the robustness of this consistency despite varying soil types, precipitation patterns, and seasonal conditions, with consistency observed in 75% of environments (6 of 8 site-years).

However, the differential responses between yield and GPC reveal important considerations for research design. While yield response patterns showed strong consistency between N rates, GPC responses diverged significantly. Yield responses reach a maximum at both 100 and 200 kg N ha⁻¹ with similar relative timing effects. Meanwhile, GPC continued to respond linearly to increased N application, increasing from 12.4% to 14.2% when the N input was doubled. This differential response indicates that consistency between the two rates may be parameter-specific (e.g. for grain yield) rather than universal across all wheat quality traits (Dick et al., 2016; Sieling and Kage, 2021).

From a physiological perspective, the consistency in timing responses likely stems from similar N uptake and allocation mechanisms across application rates. Metabolic pathways governing grain filling processes respond proportionally to N availability. Wheat plants demonstrate similar relative responses to timing treatments regardless of total N supply (Hawkesford, 2014; Fang et al., 2024). Post-anthesis N remobilization further explains the physiological basis for predictive relationships. N remobilization patterns from vegetative tissues to grains respond consistently to timing treatments across application rates, suggesting fundamental physiological constraints that govern timing responses independent of N rate. The relative efficiency of different timing strategies remains stable regardless of total N supply. This indicates fundamental physiological constraints that govern timing responses independent of N rate (Masclaux-Daubresse et al., 2010; Kichey et al., 2007).

### Application timing effects on crop performance

The physiological synchronization of N availability with wheat developmental stages represents the cornerstone of precision fertilizer management. Our study demonstrates that application timing fundamentally governs the yield-protein relationship through distinct physiological mechanisms operating at different growth stages. The critical timing window for maximum N uptake efficiency occurred between tillering initiation and flag leaf emergence (30–90 GDDs), when wheat plants exhibit peak root activity, maximum transpiration rates, and the greatest N demand for biomass accumulation (Allard et al., 2013; Hawkesford, 2014; Ding et al., 2023). During this developmental phase, N applications directly enhance yield components including spikelet number per head, spike density per m², and individual kernel weight contributing to increased yield, through optimized resource allocation during critical periods (Schulz et al., 2015).

In contrast, applications beyond 90 GDD might encounter physiological constraints including reduced root surface area, declining transpiration rates, and limited translocation capacity from vegetative tissues to developing grains (Sakuma and Schnurbusch, 2020). This creates a metabolic bottleneck that may explain the 25% yield penalty observed at 120 GDD timing across both N rates. Application at 60 GDD achieved similar yield but lower GPC, indicating insufficient delay for protein enhancement, while 120 GDD applications maximized GPC but incurred significant yield penalties. The 90 GDD timing thus represents a physiological window where crop developmental stage, N uptake capacity, and metabolic partitioning may align to optimize both agronomic objectives simultaneously.

Environmental variability amplifies these timing effects through complex interactions between delayed N applications and weather dependent physiological constraints. Drought conditions reduce root hydraulic conductivity and limit transpiration-driven nutrient transport during late applications. Conversely, excessive precipitation dilutes N concentrations and reduces efficiency in uptake. These interactions explain why the 90 GDD timing demonstrated superior stability across 75% of environments (6 of 8 site-years). This contrasts with the highly variable performance of 120 GDD applications, where yield reductions ranged from 15-35% depending on seasonal conditions. This creates the characteristic yield-protein trade-off that defines late-season N management strategies (Ercoli et al., 2013). These timing insights provide the foundation for evaluating the comparative efficiency of single versus split application methods.

### Application efficiency and management implications

The comparative efficiency of split versus single N applications addresses a critical knowledge gap in central Great Plains wheat production. Our systematic comparison across multiple environments reveals that split applications demonstrate intermediate performance for both yield and GPC parameters, providing no clear efficiency advantages over strategically timed single applications when total N rates remain equivalent. These results reject our third hypothesis, challenging conventional assumptions that dividing N inputs enhances uptake efficiency, and instead demonstrating that application timing precision may outweighs method complexity in determining agronomic outcomes under these conditions.

The mechanistic basis for single application performances likely involves optimal synchronization between N supply and crop physiological demand, potentially minimizing soil N losses during periods of low plant uptake while preventing early-season N immobilization in soil organic matter and microbial biomass (Woodley et al., 2025). In-season applications at early to mid-season timings (30–90 GDDs) achieved equivalent yields while producing higher GPC compared to both pre-plant and split applications. This possibly reflects improved temporal alignment with crop demand that reduces losses through leaching, denitrification, and volatilization (Wang et al., 2023).

The practical implications extend beyond agronomic performance to encompass operational efficiency. Single in-season applications reduce equipment passes, minimize labor requirements, and provide flexibility in timing, enabling producers to adjust N rates according to real-time assessments of crop growth stage, weather conditions, and yield potential (Ali et al., 2025). The superior performance of single applications across 75% of environments (6 of 8 site-years) under diverse precipitation patterns, soil types, and temperature regimes suggests robust reliability under Great Plains dryland conditions.

However, it is important to acknowledge that our findings are specific to the environmental and edaphic conditions of this study non-irrigated winter wheat production systems on silt loam to clay loam soils under moderate precipitation regimes (450–650 mm annually). Split application strategies may provide greater benefits in contrasting environments, including coarse-textured soils with high leaching potential (Tedone et al., 2018), irrigated systems where fertigation enables precise synchronization of N supply with crop demand (Preza-Fontes et al., 2021), or high-rainfall environments (>800 mm annually) where early-season N losses may be substantial (Schulz et al., 2015). Additionally, split applications may offer advantages when total N rates exceed 150 kg ha⁻¹, where single applications could increase lodging risk or exceed crop uptake capacity during critical growth stages. Therefore, while our results provide evidence for the efficiency of single in-season applications under Great Plains dryland conditions, producers should consider site-specific factors including soil texture, precipitation patterns, and total N requirements when selecting application strategies.

## Conclusion

Our multi-environment study across eight site-years in the central Great Plains addressed three fundamental questions about N rate optimization, application method efficiency, and timing effects on dual yield-protein objectives in winter wheat production systems. Relative response patterns at 100 kg N ha⁻¹ were consistent with those at 200 kg N ha⁻¹ across 75% of environments. This supports our first hypothesis and suggests that timing research may be conducted efficiently at moderate rates with confidence in applicability to commercial scenarios. Application timing significantly influenced both grain yield and GPC relationships, confirming our second hypothesis: in-season applications at 90 GDD (late tillering, Feekes 5-6) achieved 4.3 Mg ha⁻¹ with 13.7% GPC compared to pre-plant applications, effectively balancing dual agronomic objectives. Contrary to our third hypothesis, split N applications provided no clear advantage over single in-season applications when total rates were equivalent. This suggests that single applications at optimized timings may achieve comparable outcomes while reducing operational complexity. These findings suggest that strategic timing precision at 90 GDD, rather than rate manipulation or application splitting, may be the primary driver of N use efficiency in central Great Plains dryland winter wheat systems. However, these recommendations are specific to non-irrigated systems under moderate precipitation regimes. Future research should investigate irrigation × timing interactions and economic analysis to further refine recommendations for sustainable wheat production systems.

## Data Availability

The raw data supporting the conclusions of this article will be made available by the authors, without undue reservation.

## References

[B1] Abiola S. O. Lacasa J. Carver B. F. Arnall B. D. Ciampitti I. A. de Oliveira Silva A. (2024). Nitrogen uptake dynamics of high and low protein wheat genotypes. Front. Plant Sci. 15. doi: 10.3389/fpls.2024.1493901, PMID: 39741682 PMC11686436

[B2] Abiola S. O. Sharry R. Bushong J. Arnall D. B. (2025). Optimizing spray technology and nitrogen sources for wheat grain protein enhancement. Agriculture 15, 812. doi: 10.3390/agriculture15080812

[B3] Alcoz M. M. Hons F. M. Haby V. A. (1993). Nitrogen fertilization timing effect on wheat production, nitrogen uptake efficiency, and residual soil nitrogen. Agron. J. 85, 1198–1203. doi: 10.2134/agronj1993.00021962008500060020x

[B4] Ali A. Jabeen N. Farruhbek R. Chachar Z. Laghari A. A. Chachar S. . (2025). Enhancing nitrogen use efficiency in agriculture by integrating agronomic practices and genetic advances. Front. Plant Sci. 16. doi: 10.3389/fpls.2025.1543714, PMID: 40161228 PMC11951869

[B5] Allard V. Martre P. Le Gouis J. (2013). Genetic variability in biomass allocation to roots in wheat is mainly related to crop tillering dynamics and nitrogen status. Eur. J. Agron. 46, 68–76. doi: 10.1016/j.eja.2012.12.004

[B6] Ballagh B. Ballagh A. Bushong J. Arnall D. B. (2025). The effect of nitrogen fertilizer placement and timing on winter wheat grain yield and protein concentration. Agronomy 15, 1890. doi: 10.3390/agronomy15081890

[B7] Barneix A. J. (2007). Physiology and biochemistry of source-regulated protein accumulation in the wheat grain. J. Plant Physiol. 164, 581–590. doi: 10.1016/j.jplph.2006.03.009, PMID: 16690166

[B8] Bates D. Mächler M. Bolker B. Walker S. (2015). Fitting linear mixed-effects models using lme4. J. Stat. Software 67, 1–48. doi: 10.18637/jss.v067.i01

[B9] Bushong J. T. Arnall D. B. Raun W. R. (2014). Effect of preplant irrigation, nitrogen fertilizer application timing, and phosphorus and potassium fertilization on winter wheat grain yield and water use efficiency. Int. J. Agron. 2014, 312416. doi: 10.1155/2014/312416

[B10] Cassman K. G. Dobermann A. Walters D. T. (2002). Agroecosystems, nitrogen-use efficiency, and nitrogen management. AMBIO 31, 132–140. doi: 10.1579/0044-7447-31.2.132, PMID: 12078002

[B11] Dick C. D. Thompson N. M. Epplin F. M. Arnall D. B. (2016). Managing late-season foliar nitrogen fertilization to increase grain protein for winter wheat. Agron. J. 108, 2329–2338. doi: 10.2134/agronj2016.02.0106

[B12] Ding Y. G. Zhang X. B. Quan M. A. Li F. J. Tao R. R. Min Z. H. U. . (2023). Tiller fertility is critical for improving grain yield, photosynthesis, and nitrogen efficiency in wheat. J. Integr. Agric. 22, 2054–2066. doi: 10.1016/j.jia.2022.10.005

[B13] Ercoli L. Masoni A. Pampana S. Mariotti M. Arduini I. (2013). As durum wheat productivity is affected by nitrogen fertilisation management in Central Italy. Eur. J. Agron. 44, 38–45. doi: 10.1016/j.eja.2012.08.005

[B14] Fang L. Struik P. C. Girousse C. Yin X. Martre P. (2024). Source–sink relationships during grain filling in wheat in response to various temperature, water deficit, and nitrogen deficit regimes. J. Exp. Bot. 75, 6563–6578. doi: 10.1093/jxb/erae310, PMID: 39021198 PMC11522979

[B15] FAOSTAT (2018). Wheat Production Statistics. Available online at: http://www.fao.org/faostat/en/data/QC (Accessed July 1, 2025).

[B16] Food and Agriculture Organization of the United Nations (2015). FAO statistical Pocketbook…: World Food and Agriculture. (Rome, Italy: FAO), 28.

[B17] Fox J. Weisberg S. Adler D. Bates D. Baud-Bovy G. Ellison S. . (2012). Package ‘car’ Vol. 16 (Vienna: R Foundation for Statistical Computing), 333.

[B18] Hawkesford M. J. (2014). Reducing the reliance on nitrogen fertilizer for wheat production. J. Cereal Sci. 59, 276–283. doi: 10.1016/j.jcs.2013.12.001, PMID: 24882935 PMC4026125

[B19] Hothorn T. Bretz F. Westfall P. (2008). Simultaneous inference in general parametric models. Biometr. J. 50, 346–363. doi: 10.1002/bimj.200810425, PMID: 18481363

[B20] Hu C. Sadras V. O. Lu G. Zhang P. Han Y. Liu L. . (2021). A global meta-analysis of split nitrogen application for improved wheat yield and grain protein content. Soil Tillage Res. 213, 105111. doi: 10.1016/j.still.2021.105111

[B21] Huang S. He P. Jia L. Ding W. Ullah S. Zhao R. . (2021). Improving nitrogen use efficiency and reducing environmental cost with long-term nutrient expert management in a summer maize-winter wheat rotation system. Soil Tillage Res. 213, 105117. doi: 10.1016/j.still.2021.105117

[B22] Kichey T. Hirel B. Heumez E. Dubois F. Le Gouis J. (2007). In winter wheat (Triticum aestivum L.), post-anthesis nitrogen uptake and remobilisation to the grain correlates with agronomic traits and nitrogen physiological markers. Field Crop Res. 102, 22–32. doi: 10.1016/j.fcr.2007.01.002

[B23] Kuznetsova A. Brockhoff P. B. Christensen R. H. B. (2017). lmerTest package: tests in linear mixed effects models. J. Stat. Software 82. doi: 10.18637/jss.v082.i13

[B24] Li W. Han M. Pang D. Chen J. Wang Y. Dong H. . (2022). Characteristics of lodging resistance of high-yield winter wheat as affected by nitrogen rate and irrigation managements. J. Integrative Agriculture. 21 (5), 1290–1309. doi: 10.1016/S2095-3119(20)63566-3

[B25] Ma Q. Wang M. Zheng G. Yao Y. Tao R. Zhu M. . (2021). Twice-split application of controlled-release nitrogen fertilizer met the nitrogen demand of winter wheat. Field Crops Res. 267, 108163. doi: 10.1016/j.fcr.2021.108163

[B26] Masclaux-Daubresse C. Daniel-Vedele F. Dechorgnat J. Chardon F. Gaufichon L. Suzuki A. (2010). Nitrogen uptake, assimilation and remobilization in plants: challenges for sustainable and productive agriculture. Ann. Bot. 105, 1141–1157. doi: 10.1093/aob/mcq028, PMID: 20299346 PMC2887065

[B27] McPherson R. A. Fiebrich C. A. Crawford K. C. Kilby J. R. Grimsley D. L. Martinez J. E. . (2007). Statewide monitoring of the mesoscale environment: A technical update on the Oklahoma Mesonet. J. Atmos. Oceanic Tech. 24, 301–321. doi: 10.1175/JTECH1976.1

[B28] Miller E. C. Bushong J. T. Raun W. R. Abit M. J. M. Arnall D. B. (2017). Predicting early season nitrogen rates of corn using indicator crops. Agron. J. 109, 2863–2870. doi: 10.2134/agronj2016.09.0519

[B29] Mohammed Y. A. Kelly J. Chim B. K. Rutto E. Waldschmidt K. Mullock J. . (2013). Nitrogen fertilizer management for improved grain quality and yield in winter wheat in Oklahoma. J. Plant Nutr. 36, pp.749–pp.761. doi: 10.1080/01904167.2012.754039

[B30] Noulas C. Torabian S. Qin R. (2023). Crop nutrient requirements and advanced fertilizer management strategies. Agronomy 13, 2017. doi: 10.3390/agronomy13082017

[B31] Oklahoma Mesonet Station (2012). Degree-day Heat Unit Calculator. Available online at: https://www.mesonet.org/images/site/Degree%20day%20Heat%20Unit%20Calculator%20text(1).pdf (Accessed June 30, 2025).

[B32] Pan W. L. Kidwell K. K. McCracken V. A. Bolton R. P. Allen M. (2020). Economically optimal wheat yield, protein and nitrogen use component responses to varying N supply and genotype. Front. Plant Sci. 10. doi: 10.3389/fpls.2019.01790, PMID: 32158450 PMC7052120

[B33] Preza-Fontes G. Pittelkow C. M. Greer K. D. Bhattarai R. Christianson L. E. (2021). Split‐nitrogen application with cover cropping reduces subsurface nitrate losses while maintaining corn yields. J. Environ. Qual. 50. 1408–1418. doi: 10.1002/jeq2.20283, PMID: 34390507

[B34] Sakuma S. Schnurbusch T. (2020). Of floral fortune: tinkering with the grain yield potential of cereal crops. New Phytol. 225, 1873–1882. doi: 10.1111/nph.16189, PMID: 31509613

[B35] Schulz R. Makary T. Hubert S. Hartung K. Gruber S. Donath S. . (2015). Is it necessary to split nitrogen fertilization for winter wheat? On-farm research on Luvisols in South-West Germany. J. Agric. Sci. 153, pp.575–pp.587. doi: 10.1017/S0021859614000288, PMID: 26063931 PMC4453072

[B36] Sieling K. Kage H. (2021). Apparent fertilizer N recovery and the relationship between grain yield and grain protein concentration of different winter wheat varieties in a long-term field trial. Eur. J. Agron. 124, 126246. doi: 10.1016/j.eja.2021.126246

[B37] Singh S. Kaur J. Ram H. Singh J. Kaur S. (2023a). Agronomic bio-fortification of wheat (Triticum aestivum L.) to alleviate zinc deficiency in human being. Rev. Environ. Sci. Bio/Technology 22, 505–526. doi: 10.1007/s11157-023-09653-4, PMID: 37234132 PMC10134721

[B38] Singh R. Sawatzky S. Thomas M. Akin S. Raun W. R. Zhang H. . (2024). Micronutrients concentration and content in corn as affected by nitrogen, phosphorus, and potassium fertilization. Agrosystems Geosciences Environ. 7, e20568. doi: 10.1002/agg2.20568

[B39] Singh R. Sawatzky S. K. Thomas M. Akin S. Zhang H. Raun W. . (2023b). Nitrogen, phosphorus, and potassium uptake in rain-fed corn as affected by NPK fertilization. Agronomy 13, 1913. doi: 10.3390/agronomy13071913

[B40] Snyder C. S. (2017). Enhanced nitrogen fertiliser technologies support the ‘4R’concept to optimise crop production and minimise environmental losses. Soil Res. 55, 463–472. doi: 10.1071/SR16335

[B41] Souza J. L. B. Antonangelo J. A. de Oliveira Silva A. Reed V. Arnall B. (2022). Recovery of grain yield and protein with fertilizer application post nitrogen stress in winter wheat (Triticum aestivum L.). Agronomy 12, 2024. doi: 10.3390/agronomy12092024

[B42] Szczepaniak W. Grzebisz W. Potarzycki J. (2022). Yield predictive worth of pre-flowering and post-flowering indicators of nitrogen economy in high yielding winter wheat. Agron. 13, 122. doi: 10.3390/agronomy13010122

[B43] Tedone L. Ali S. A. Verdini L. De Mastro G. (2018). Nitrogen management strategy for optimizing agronomic and environmental performance of rainfed durum wheat under Mediterranean climate. J. cleaner production 172, 2058–2074. doi: 10.1016/j.jclepro.2017.11.215

[B44] Triboi E. Martre P. Girousse C. Ravel C. Triboi-Blondel A. M. (2006). Unravelling environmental and genetic relationships between grain yield and nitrogen concentration for wheat. Eur. J. Agron. 25, 108–118. doi: 10.1016/j.eja.2006.04.004

[B45] USDA-Agricultural Marketing Service (USDA-AIMS) . (2024). Discounts for Wheat, Barley, and Oats. chrome-extension://efaidnbmnnnibpcajpcglclefindmkaj/https://www.fsa.usda.gov/sites/default/files/documents/2024-Discounts-for-Wheat-Barley-and-Oats.pdf (Accessed October 19, 2025).

[B46] Wang J. Sha Z. Zhang J. Qin W. Xu W. Goulding K. . (2023). Improving nitrogen fertilizer use efficiency and minimizing losses and global warming potential by optimizing applications and using nitrogen synergists in a maize-wheat rotation. Agriculture Ecosyst. Environ. 353, 108538. doi: 10.1016/j.agee.2023.108538

[B47] Wang D. Xu Z. Zhao J. Wang Y. Yu Z. (2011). Excessive nitrogen application decreases grain yield and increases nitrogen loss in a wheat–soil system. Acta Agriculturae Scandinavica Section B-Soil Plant Sci. 61, 681–692. doi: 10.1080/09064710.2010.534108

[B48] Warren J. Zhang H. Arnall B. Bushong J. Raun B. Penn C. . (2017). Oklahoma Soil Fertility Handbook (Stillwater, OK, USA: Oklahoma Cooperative Extension Service). Available online at: https://extension.okstate.edu/fact-sheets/oklahoma-soil-fertility-handbook-full.html (Accessed June 30, 2025).

[B49] Wickham H. (2016). “ Data analysis,” in ggplot2: Elegant graphics for data analysis ( Springer International Publishing, Cham), 189–201. doi: 10.1007/978-3-319-24277-4_9

[B50] Wickham H. Francois R. Henry L. Müller K. (2020). Dplyr: A Grammar of Data Manipulation. R Package Version 0.8.4. Available online at: https://CRAN.R-project.org/package=dplyr (Accessed October 26, 2025).

[B51] Woodley A. L. Drury C. F. Yang X. Y. Phillips L. A. Reynolds W. D. Calder W. . (2025). Delaying application and injecting nitrogen fertilizer with urease and nitrification inhibitors decreased nitrous oxide emissions and enhanced corn yields. J. Environ. Qual. 54, 925–933. doi: 10.1002/jeq2.70044, PMID: 40474351 PMC12431951

[B52] Wu W. Wang Y. Xu H. Liu M. Xue C. (2025). Enhancing wheat yield and quality through late-season foliar nitrogen application: A global meta-analysis. Agronomy 15, 1058. doi: 10.3390/agronomy15051058

[B53] Zhao H. Zhang L. Kirkham M. B. Welch S. M. Nielsen-Gammon J. W. Bai G. . (2022). US winter wheat yield loss attributed to compound hot-dry-windy events. Nat. Commun. 13, p.7233. doi: 10.1038/s41467-022-34947-6, PMID: 36433980 PMC9700680

