# Supplementary material for: Split nitrogen applications provide no benefit over a single well timed application in rainfed winter wheat
Source: Front Plant Sci. 2025 Nov 6;16:1698494. doi: 10.3389/fpls.2025.1698494 (PMC12632810; doi:10.3389/fpls.2025.1698494)
Supplement: Supplementary file 1 [file DataSheet1.docx]

**
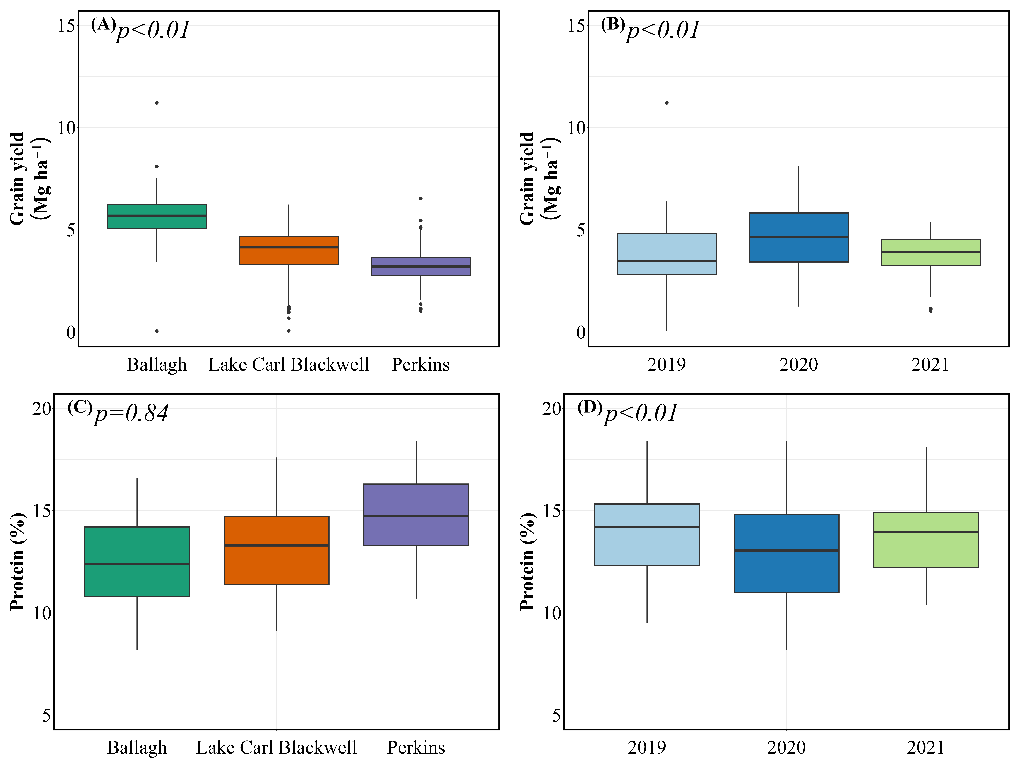
**

**Figure 1:** The homogeneity of variance for grain yield (Mg ha⁻¹) and protein (%) across different sites and years (*p<0.05).*

| **Growing seasons** | **Sites** |  | **0** | | **30** | | **60** | | **90** | | **120** | |
| --- | --- | --- | --- | --- | --- | --- | --- | --- | --- | --- | --- | --- |
|  |  | **N rates** | **Yield** | **GPC** | **Yield** | **GPC** | **Yield** | **GPC** | **Yield** | **GPC** | **Yield** | **GPC** |
| 2018-2019 | Ballagh | 0 | 4.5 | 11.0c | 4.5 | 11.0c | 4.5b | 11.0c | 4.5 | 11.0b | 4.5b | 11.0c |
|  |  | 100 | 7.0 | 12.7b | 5.2 | 12.6b | 5.1ab | 13.4b | 5.5 | 14.1a | 5.4a | 15.1b |
|  |  | 200 | 5.1 | 14.6a | 5.5 | 14.5a | 5.3a | 15.0a | 4.2 | 15.7a | 5.2a | 16.3a |
|  |  | *p-value* | *0.14* | *<0.01* | *0.12* | *<0.01* | *<0.05* | *<0.01* | *0.49* | *<0.01* | *0.06* | *<0.01* |
|  | LCB | 0 | 0.8b | 11.2 | 0.8b | 11.2b | 0.8b | 11.2b | 0.8b | 11.2b | 0.8 | 11.2 |
|  |  | 100 | 3.6a | 11.4 | 3.5a | 13.1ab | 3.7a | 13.2ab | 2.7a | 14.4ab | 1.7 | 10.1 |
|  |  | 200 | 2.6ab | 12.2 | 3.3a | 15.4a | 3.8a | 15.6a | 3.2a | 15.9a | 1.3 | 10.2 |
|  |  | *p-value* | *<0.01* | *0.83* | *<0.01* | *<0.05* | *<0.01* | *<0.05* | *<0.01* | *<0.05* | *0.39* | *0.51* |
|  | Perkins | 0 | 1.6b | 12.8c | 1.6b | 12.8c | 1.6b | 12.8c | 1.6b | 12.8c | 1.6b | 12.8c |
|  |  | 100 | 3.3a | 14.0b | 3.5a | 16.9b | 2.9a | 15.1a | 3.0a | 15.5b | 2.6a | 16.2b |
|  |  | 200 | 3.3a | 15.9a | 3.3a | 18.2a | 3.2a | 16.8a | 3.3a | 17.9a | 2.8a | 17.9a |
|  |  | *p-value* | *<0.01* | *<0.01* | *<0.01* | *<0.01* | *<0.01* | *<0.01* | *<0.01* | *<0.01* | *<0.01* | *<0.01* |
| 2019-2020 | Ballagh | 0 | 4.2b | 8.5c | 4.2b | 8.5c | 4.2b | 8.5b | 4.2b | 8.4c | 4.2 | 8.5c |
|  |  | 100 | 6.3a | 10.1b | 6.1a | 10.5b | 6.3a | 10.3ab | 6.4a | 10.3b | 5.4 | 13.1b |
|  |  | 200 | 6.8a | 12.0a | 6.4a | 12.2a | 6.3a | 11.8a | 6.6a | 11.8a | 5.0 | 14.8a |
|  |  | *p-value* | *<0.01* | *<0.01* | *<0.01* | *<0.01* | *<0.01* | *<0.01* | *<0.05* | *<0.01* | *0.21* | *<0.01* |
|  | LCB | 0 | 2.1b | 9.5c | 2.1b | 9.5c | 2.1b | 9.5c | 2.1b | 9.5c | 2.1b | 9.5c |
|  |  | 100 | 4.6a | 10.9b | 4.3a | 11.3b | 4.8a | 11.4b | 5.0a | 12.5b | 4.0a | 15.1b |
|  |  | 200 | 5.6a | 13.6a | 5.3a | 13.3a | 5.5a | 13.8a | 5.5a | 14.5a | 4.2a | 16.3c |
|  |  | *p-value* | *<0.01* | *<0.01* | *<0.01* | *<0.01* | *<0.01* | *<0.01* | *<0.01* | *<0.01* | *<0.01* | *<0.01* |
|  | Perkins | 0 | 2.4b | 12.9c | 2.4b | 12.9c | 2.4b | 12.9c | 2.4 | 12.9b | 2.4 | 12.9b |
|  |  | 100 | 3.6b | 14.0b | 3.4ab | 14.3b | 4.6a | 15.1b | 3.0 | 15.5ab | 2.6 | 16.2a |
|  |  | 200 | 5.4a | 15.9a | 3.7a | 16.1a | 3.2ab | 16.8a | 3.4 | 17.2a | 2.8 | 17.9a |
|  |  | *p-value* | *<0.01* | *<0.01* | *<0.05* | *<0.01* | *<0.01* | *<0.01* | *0.17* | *<0.01* | *0.77* | *<0.01* |
|  | LCB | 0 | 3.5b | 11.5c | 3.5b | 11.5b | 3.5b | 11.5b | 3.5b | 11.5c | 3.5 | 11.5b |
| 2020-2021 |  | 100 | 4.5a | 13.0b | 4.3ab | 13.8a | 4.4ab | 14.0a | 4.4a | 14.8b | 3.7 | 14.6a |
|  |  | 200 | 4.4a | 14.6a | 4.9a | 14.5a | 4.8a | 14.9a | 4.0a | 15.8a | 3.9 | 15.5a |
|  |  | *p-value* | *<0.01* | *<0.01* | *<0.01* | *<0.01* | *<0.01* | *<0.01* | *0.06* | *<0.01* | *0.24* | *<0.01* |
|  | Perkins | 0 | 1.1b | 10.9b | 1.1c | 11.0b | 1.1c | 11.0b | 1.1b | 11.0c | 1.1c | 11.0c |
|  |  | 100 | 3.1a | 12.9ab | 3.2b | 11.7b | 3.9b | 11.7b | 4.2a | 12.5b | 1.8b | 16.6b |
|  |  | 200 | 3.5a | 14.4a | 4.2c | 14.0a | 4.7a | 14.0a | 4.3a | 15.9a | 2.1a | 17.7a |
|  |  | *p-value* | *<0.01* | *<0.05* | *<0.01* | *<0.01* | *<0.01* | *<0.01* | *<0.01* | *<0.01* | *<0.01* | *<0.01* |

**Table 1.** Grain yield and grain protein concentration (%) responses to N application rates (0, 100, 200 kg N ha^-1^) at different growing degree days across three locations (Ballagh, LCB, and Perkins) during the 2018-2019, 2019-2020, and 2020-2021 growing seasons.

*Different letters represent significant statistical difference (Tukey, HSD) at the 0.05 probability level.*

**Table 2**. Grain yield and grain protein concentration (%) responses to N application timings (0, 30, 60, 90, 120 GDDs) for two N rates of applications across three locations (Ballagh, LCB, and Perkins) during 2018-2019, 2019-2020, and 2020-2021 growing seasons.

| **Years** | **Sites** |  | **50-50** | | **100** | | **200** | | |
| --- | --- | --- | --- | --- | --- | --- | --- | --- | --- |
|  |  | **Timing** | **Yield** | **GPC** | **Yield** | **GPC** | | **Yield** | **GPC** |
| 2018-2019 | Ballagh | 0 | 7.0a | 12.7b | 7.0 | 12.7ab | | 5.1 | 14.6b |
|  |  | 30 | 5.7 | 12.5b | 6.2 | 12.6b | | 5.5 | 14.5b |
|  |  | 60 | 4.9 | 13.4a | 5.1 | 13.4ab | | 5.3 | 14.9b |
|  |  | 90 | 5.0 | 13.7a | 5.5 | 14.1ab | | 4.2 | 15.7ab |
|  |  | 120 | 5.9 | 13.8a | 5.4 | 15.1a | | 5.2 | 16.2a |
|  |  | *p-value* | *0.18* | *0.08* | *0.36* | *<0.05* | | *0.68* | *<0.01* |
|  | LCB | 0 | 3.6 | 11.4ab | 3.6a | 11.4bc | | 2.6b | 12.2bc |
|  |  | 30 | 3.2 | 10.6ab | 3.5a | 13.1ab | | 3.3a | 15.4ab |
|  |  | 60 | 3.4 | 10.9ab | 3.7a | 13.2ab | | 3.8a | 15.6a |
|  |  | 90 | 3.2 | 11.9a | 2.7ab | 14.4a | | 3.2a | 15.9a |
|  |  | 120 | 2.4 | 10.1b | 1.7b | 10.1c | | 1.3c | 10.2c |
|  |  | *p-value* | *0.45* | *<0.05* | *<0.01* | *<0.05* | | *0.08* | *<0.01* |
|  | Perkins | 0 | 3.3ab | 13.9ab | 3.3ab | 13.9d | | 3.3a | 15.9c |
|  |  | 30 | 3.6a | 14.8a | 3.5a | 16.9a | | 3.3a | 18.2a |
|  |  | 60 | 3.2ab | 13.5b | 2.9bc | 15.1c | | 3.2ab | 16.8bc |
|  |  | 90 | 2.9b | 13.7b | 3.0abc | 15.5bc | | 3.3a | 17.9ab |
|  |  | 120 | 3.0b | 14.1ab | 2.6c | 16.2ab | | 2.8b | 18.0ab |
|  |  | *p-value* | *<0.05* | *<0.05* | *<0.05* | *<0.01* | | *<0.05* | *<0.01* |
| 2019-2020 | Ballagh | 0 | 6.3a | 10.1ab | 6.3 | 10.1b | | 6.8a | 12.0b |
|  |  | 30 | 6.3a | 9.9b | 6.1 | 10.5b | | 6.4a | 12.2b |
|  |  | 60 | 5.9b | 10.0b | 6.3 | 10.3b | | 6.3a | 11.8b |
|  |  | 90 | 6.4a | 10.0b | 6.4 | 10.3b | | 6.6a | 11.8b |
|  |  | 120 | 6.3a | 11.8a | 5.4 | 13.1a | | 5.0b | 14.8a |
|  |  | *p-value* | *<0.01* | *<0.05* | *0.29* | *<0.01* | | *0.06* | *<0.01* |
|  | LCB | 0 | 4.6 | 10.9b | 4.6 | 10.9b | | 5.5a | 13.6b |
|  |  | 30 | 4.8 | 11.6ab | 4.3 | 11.3b | | 5.3a | 13.3b |
|  |  | 60 | 4.2 | 10.7b | 4.8 | 11.4b | | 5.5a | 13.8b |
|  |  | 90 | 4.3 | 11.6ab | 5.0 | 12.5b | | 5.5a | 14.5ab |
|  |  | 120 | 4.3 | 13.0a | 4.0 | 15.1a | | 4.2b | 16.3a |
|  |  | *p-value* | *0.65* | *<0.05* | *0.12* | *<0.01* | | *<0.01* | *<0.01* |
|  | Perkins | 0 | 3.7a | 13.9ab | 3.7b | 13.9d | | 5.4a | 15.9b |
|  |  | 30 | 3.6a | 14.8a | 3.4bc | 14.3cd | | 3.7b | 16.1ab |
|  |  | 60 | 3.2b | 13.5b | 4.6a | 15.1bc | | 3.2bc | 16.8ab |
|  |  | 90 | 3.0b | 13.7b | 3.0cd | 15.5ab | | 3.4bc | 17.2ab |
|  |  | 120 | 3.0b | 14.1ab | 2.6d | 16.2a | | 2.8c | 17.9a |
|  |  | *p-value* | *<0.01* | *<0.01* | *<0.01* | *<0.01* | | *<0.01* | *<0.05* |
|  | LCB | 0 | 4.5 | 13.0ab | 4.5 | 13.0b | | 4.4a | 14.6b |
| 2020-2021 |  | 30 | 4.6 | 12.9b | 4.3 | 13.8ab | | 4.9a | 14.5b |
|  |  | 60 | 4.7 | 13.5a | 4.4 | 14.0ab | | 4.8a | 14.9ab |
|  |  | 90 | 4.5 | 13.8a | 4.4 | 14.8a | | 4.0a | 15.8a |
|  |  | 120 | 4.5 | 14.2a | 3.7 | 14.6ab | | 3.9b | 15.4ab |
|  |  | *p-value* | *0.87* | *<0.05* | *0.25* | *<0.05* | | *<0.05* | *<0.05* |
|  | Perkins | 0 | 3.1ab | 12.9ab | 3.1b | 12.9b | | 3.5b | 14.4b |
|  |  | 30 | 2.8b | 11.7b | 3.2b | 11.7b | | 4.2ab | 14.0b |
|  |  | 60 | 3.2ab | 12.2b | 3.9ab | 11.7b | | 4.7a | 14.0b |
|  |  | 90 | 3.4a | 11.6b | 4.2a | 12.5b | | 4.3ab | 15.9ab |
|  |  | 120 | 2.3c | 14.8a | 1.8c | 16.6a | | 2.0c | 17.7a |
|  |  | *p-value* | *<0.01* | *<0.01* | *<0.01* | *<0.01* | | *<0.01* | *<0.01* |

*Different letters represent significant statistical difference (Tukey, HSD) at the 0.05 probability level.*

**Table 3**. Grain yield and grain protein concentration responses to nitrogen application timing and methods across three locations during 2018-2021 growing seasons. Pre-applied, in-season, and split-applied treatments were evaluated at 100 kg N ha⁻¹ across four timing intervals (30, 60, 90, and 120 growing degree days).

| **Years** | **Sites** |  | **30** | | **60** | | **90** | | **120** | |
| --- | --- | --- | --- | --- | --- | --- | --- | --- | --- | --- |
|  |  | **N rates** | **Yield** | **GPC** | **Yield** | **GPC** | **Yield** | **GPC** | **Yield** | **GPC** |
| 2018-2019 | Ballagh | Pre-applied | 7.0 | 12.7 | 7.0 | 12.7 | 7.0 | 12.7 | 7.0 | 12.7b |
|  |  | In-season | 5.2 | 12.6 | 5.1 | 13.4 | 5.5 | 14.1 | 5.4 | 15.1a |
|  |  | Split-applied | 5.7 | 12.5 | 4.9 | 13.4 | 5.0 | 13.7 | 5.9 | 13.8b |
|  |  | *p-value* | *0.36* | *0.87* | *0.19* | *0.12* | *0.33* | *0.24* | *0.46* | *0.01* |
|  | LCB | Pre-applied | 3.6 | 11.4ab | 3.6 | 11.4ab | 3.6a | 11.4b | 3.6 | 11.4a |
|  |  | In-season | 3.5 | 13.1a | 3.7 | 13.2a | 2.7b | 14.4a | 1.7 | 10.1b |
|  |  | Split-applied | 3.2 | 10.5b | 3.4 | 10.9b | 3.2ab | 11.9b | 2.4 | 10.1b |
|  |  | *p-value* | *0.66* | *<0.05* | *0.29* | *<0.05* | *<0.01* | *<0.01* | *0.14* | *<0.01* |
|  | Perkins | Pre-applied | 3.3 | 14.0c | 3.3a | 13.9b | 3.3 | 14.0b | 3.3a | 14.0b |
|  |  | In-season | 3.5 | 16.9a | 2.9b | 15.1a | 3.0 | 15.5a | 2.6b | 16.2a |
|  |  | Split-applied | 3.6 | 14.8b | 3.2a | 13.5b | 3.0 | 13.7b | 3.0ab | 14.1b |
|  |  | *p-value* | *0.22* | *<0.01* | *0.07* | *<0.01* | *0.18* | *<0.05* | *<0.01* | *<0.01* |
| 2019-2020 | Ballagh | Pre-applied | 6.3 | 10.1 | 0.26 | 10.1 | 6.3 | 10.1 | 6.3 | 10.1b |
|  |  | In-season | 6.1 | 10.5 | 6.3 | 10.3 | 6.4 | 10.3 | 5.4 | 13.1a |
|  |  | Split-applied | 6.3 | 10.0 | 5.9 | 10.0 | 6.4 | 10.0 | 6.3 | 11.8ab |
|  |  | *p-value* | *0.57* | *0.17* | *0.68* | *0.85* | *0.73* | *0.73* | *0.24* | *<0.01* |
|  | LCB | Pre-applied | 4.6 | 10.8 | 4.6 | 10.9 | 4.6b | 10.9c | 4.6a | 10.9c |
|  |  | In-season | 4.3 | 11.3 | 4.8 | 11.3 | 5.0a | 12.5a | 4.0b | 15.1a |
|  |  | Split-applied | 4.8 | 11.6 | 4.2 | 10.7 | 4.3b | 11.6b | 4.3ab | 13.0ab |
|  |  | *p-value* | *0.61* | *0.66* | *0.55* | *0.58* | *0.08* | *<0.01* | *<0.01* | *<0.01* |
|  | Perkins | Pre-applied | 3.7 | 13.9b | 3.6b | 13.9b | 3.7a | 14.0b | 3.7a | 14.0b |
|  |  | In-season | 3.4 | 14.3a | 4.6a | 15.1a | 3.0b | 15.5a | 2.6c | 16.2a |
|  |  | Split-applied | 3.6 | 14.8a | 3.2b | 13.5b | 3.0b | 13.7b | 3.0b | 14.1b |
|  |  | *p-value* | *0.61* | *0.06* | *<0.01* | *<0.01* | *<0.01* | *<0.01* | *<0.01* | *<0.01* |
|  | LCB | Pre-applied | 4.5 | 13.0 | 4.5 | 13.0 | 4.5 | 13.0b | 4.5a | 13.0b |
| 2020-2021 |  | In-season | 4.3 | 13.8 | 4.4 | 14.0 | 4.4 | 14.8a | 3.7b | 14.6a |
|  |  | Split-applied | 4.6 | 12.9 | 4.7 | 13.5 | 4.5 | 13.8ab | 4.5a | 14.2a |
|  |  | *p-value* | *0.77* | *0.47* | *0.43* | *0.24* | *0.90* | *<0.01* | *<0.05* | *<0.01* |
|  | Perkins | Pre-applied | 3.1a | 12.9 | 3.1b | 12.3 | 3.1b | 12.9 | 3.1a | 12.9b |
|  |  | In-season | 3.2a | 11.7 | 3.9a | 11.7 | 4.2a | 12.5 | 1.8b | 16.6a |
|  |  | Split-applied | 2.8b | 11.7 | 3.2b | 12.2 | 3.4ab | 11.6 | 2.3b | 14.8ab |
|  |  | *p-value* | *<0.05* | *0.22* | *<0.01* | *0.46* | *<0.05* | *0.31* | *<0.01* | *<0.05* |

*Different letters represent significant statistical difference (Tukey, HSD) at the 0.05 probability level.*
